# Hospital and Institutional News

**Published:** 1912-06-29

**Authors:** 


### HOSPITAL AND INSTITUTIONAL NEWS.

### THE KING'S VISIT TO BRISTOL.

Soon after The Hospital goes to press this week their Majesties the King and Queen will visit the King Edward Memorial Infirmary at Bristol, which is the finished outcome of what was known at first as the new surgical block of the Bristol Royal Infirmary. In order that our readers may accompany, so far as the art of literature allows, the King and Queen on their tour of inspection, we publish a special interview with Mr. W. E. Budgett, Secretary and House Governor of the Royal Infirmary, which contains not merely an exhaustive and technical description of the new infirmary, and of the six theatres which form its most characteristic unit, but also gives Sir George White's personal explanation of the very original plan by which the great undertaking has been financed. With Mr. Budgett's description on the one hand, and Sir George White's authoritative statement of his policy as the hospital's president on the other, we place in the hands of our readers the best substitute for a personal visit which pen, paper, and illustration under expert direction can afford. We are sure that hospital workers generally will not fail to appreciate Sir George White's personal statement and Mr. Budgett's unfailing communicativeness.

### RUSKIN'S BIOGRAPHER AND FLORENCE NIGHTINGALE.

Mr. E. T. Cook, the well-known biographer of Ruskin, whose knighthood was announced on Monday last, and whose edition of "Ruskin" is a monument of painstaking and care, will probably, so we are informed from a reliable source, compile the biography of Florence Nightingale. The qualifications which might recommend Sir E. T. Cook for such a task are obvious to all; and it is no discourtesy to suggest that the Nightingale family would probably be wise to choose a biographer outside its own immediate circle. Journalism in its more responsible aspects is no bad training for biography; it means, at any rate in Sir Edward's case, a valuable experience of all sorts of people which should be the ideal biographer's best gift. It means also a love, born of necessity, for sifting facts and for remembering gossip, while engendering responsibility and discrimination concerning them; in short, it helps to produce all those qualities which, coupled with admiration for the selected subject, helped to make Boswell a prince of literary portrait-painters. In addition to these qualities Sir Edward Cook can claim genuine scholarship, of which a first in Greats is indisputable evidence. Taking these facts at their face value, he would appear to lack only one calculable quality for this task—namely, long-continued companionship with the subject of his suggested labours. It is impossible to estimate this as unimportant, but all the hospital world will feel that to dwell on his other qualifications would be appropriate if we could soon state officially that they are to be applied in the artistic preservation of Florence Nightingale's life and work.

### A WORD OF WARNING TO THE PORTSMOUTH HEALTH COMMITTEE.

The Portsmouth Health Committee has now decided to extend the Infectious Diseases Hospital. Two blocks are proposed, each of which would contain twenty beds and be so arranged as to admit anyway some sort of classification. The buildings are to be designed by the Borough Engineer, and certain members of the Health Committee are visiting various centres, including Leeds and Bristol, to see what is being done in progressive quarters. We hope that at the latter city the Committee may find its way to the City Hospital at Ham Green, where, we have good reason to understand, the bed isolation methods are most up to date, and on the first convenient opportunity, through the courtesy of Dr. B. A. Peters, the resident medical officer, have promised to inspect those which have been inaugurated there during the past year or two. In any case all fever hospital extension schemes deserve the most watchful attention of those who desire to see the present average low standard replaced by a high one. In the provinces, as we have often pointed out, the provision of adequate bed isolation is far too frequently scamped or non-existent, and report after report comes out revealing a large amount of unnecessary cross-infection. In this connection, therefore, it is only friendly criticism to warn the Portsmouth Health Committee not to shirk their responsibility at the present point, and that such a warning is not unneeded may be judged from the fact that the new accommodation contemplated is only half the extent of that which was recommended by the medical staff. We are fully aware that there is a divergency of medical opinion to be reckoned with on certain points. But the question of extending infectious diseases hospitals is one that requires very careful watching just now.

### AMUSING SPEECHES AT A HOSPITAL'S OPENING.

We are glad to add to our previous account of its inception a notice of the opening on Thursday last week of the new Cottage Hospital at Goole, Yorkshire. This institution, which is to be known as Bartholomew Hospital, contains 24 beds; the architects are Messrs. Hill and Son, of Leeds, and the builders Ledgard and Son, of the same city. The opening was performed by Mrs. W. Warde-Aldam, of Frickley Hall, Doncaster, when Mr. H. Chapman, the chairman of the hospital committee, delivered a most interesting speech. It was in 1891 that the first hospital at Goole was opened through the generosity of Mr. W. H. Bartholomew, when a population of 12,000 required an institution. To-day that number has risen to 20,000; hence the new hospital. In a very amusing speech Mr. W. Sheepshanks, the chairman of the Aire and Calder

Navigation, which has given the site, said it was stated that the Aire and Calder Navigation had given a site for a church, and that that fact could always be counted to them for righteousness. That brought balm to his soul, because up to the present time the well-known text, "Woe unto you when all men speak well of you," could hardly be adapted to any official of Aire and Calder Navigation. When the laughter had subsided Mr. Sheepshanks remarked that, while it was a very good thing to provide a site for a church and to provide for the spiritual needs of the people, it was not enough, and so the Aire and Calder Navigation had given the site for that hospital.

### EXEMPTING MENTAL HOSPITAL STAFFS FROM THE INSURANCE ACT.

The Mental Hospitals Committee of the London County Council are applying to the Insurance Commissioners for a certificate of exemption under Clause (*b*) of Part II. of the First Schedule of the Act. The application concerns all their officers and employees who are pensionable under the Asylum Officers' Superannuation Act of 1909 and are entitled to sick pay under the Committee's regulations. They are also claiming from the Board of Trade exemption from the provisions of Part II. of the Act for those of their employees to whom Part II. of the Act would apply who are pensionable under the Asylum Officers' Superannuation Act.

### ALEXANDRA DAY.

Wednesday was Alexandra Day in London, and as we go to press it is announced that the Queen-Mother will drive round many of the districts, to encourage by her presence those ladies who have dedicated themselves on that afternoon to sell button-holes of wild roses, the proceeds from which are going to swell the funds of the hospitals. Princess Louis of Battenberg has ordered 14,000 blooms, for which she will seek purchasers around the Admiralty district, and the Duchess of Norfolk's house in St. James's Square will be the chief depôt in the West End. Actors and actresses, those tireless popularisers of other people's claims, in over forty theatres and halls will wear a wild rose during the performances. Finally we learn that already preparations are on foot to celebrate Alexandra Day throughout the Colonies in 1914.

### THE MEDICAL BENEFIT NEGOTIATIONS.

The negotiations between the Insurance Commissioners and the representatives of the medical profession over the rates of *per capita* remuneration for medical services under the Act are still shrouded in much mystery. The Chancellor's statement on Monday makes it clear that the contemplated raid on last year's realised surplus was so repugnant to important sections of his party as to be impracticable, and it has, therefore, been allowed to drop. The only other official information is that the books of certain selected practitioners in labouring-class districts are being submitted to independent audit by Sir W. Plender, in order that an impartial actuarial basis of work to be done may be arrived at. The grand flaw in this scheme is that it fails to take into account the difference between voluntary club practice and compulsory State insurance. In the former there are a considerable percentage of contributing members who, owing to distance or other causes, never trouble the club medical officer when they are ill; many of them in large towns resort to the nearest hospital, for example. In the latter case members are compelled to pay their insurance premiums, and, owing to the provisions about choice of doctor, are certain to take every advantage of "medical benefit." Furthermore, malingering is bound to be increased by this Act, just as it has been by the Compensation Acts. All these considerations must be kept in view when Sir W. Plender has completed his audit and reported the results.

### THE "PULSOCON" IN PARIS.

The Paris correspondent of the *Lancet* has done a public service by reporting to his paper the circumstances concerning the recent arrest there of Macaura, the ingenious (and unqualified) exploiter of the "pulsocon," whose lineaments a few months ago confronted every passer-by from the advertisement hoardings of London. The procedure of this fellow in Paris seems to have been modelled upon that which brought him such financial success in this country; for we are told that he attracted in a few months a numerous clientèle by lavish advertising and promises of gratuitous and marvellous cures. Fortunately, the laws of France are more stringent than those of this kingdom in many ways, and, at the instance of the Syndicate of Physicians of the Seine, Macaura was called upon to answer a charge of swindling. He was represented before the *juge d'instruction* by two counsel, but notwithstanding their efforts he was committed to prison for trial, though he was afterwards allowed bail in the sum of fifty thousand francs (£2,000). It will be instructive to learn what his eventual fate is; but it is to be feared that in the end he is likely to desert the Republic and to return to the laxer code of our happy-go-lucky system of indifference to advertised charlatanism.

### THE CAVENDISH LECTURE: A NEW DEPARTURE.

A new precedent was set this year by the President and Council of the West London Medico-Chirurgical Society in going outside the medical profession for their Cavendish Lecture. Instead of perpetuating the established custom, they conceived the excellent notion of inviting Professor Pearson to expound his views upon the medical aspect of those problems to which he devotes himself. It is true that in this exposition he advanced nothing new, because his purpose was to arouse the attention of an audience of professional men to many of whom his views are doubtless unknown; and, therefore, he had to provide elementary rather than advanced instruction. The result was that he traversed ground familiar already to those who have studied his published works. His main thesis was that by the ever-increasing efficiency of modern medicine, the survival and perpetuation of naturally weak stocks is being encouraged; and that in this way the improvement of the race by natural selection is being hindered. With his contentions for segregation of degenerates, weak minded, epileptics, and so forth most medical men will agree. But we think Professor Pearson is on much less safe ground when he deprecates efforts to lessen infantile mortality; for the causes of this mortality (even though it be really the weaklings that perish) are also the causes of much avoidable morbidity, and so of tuberculosis and the other conditions, including degeneracy, which Professor Pearson wishes to diminish.

### THE FINE ART OF HOSPITAL FUNCTIONS.

King Edward VII.'s Hospital at Cardiff has a reputation for pleasant and well-organised functions. Two took place on Wednesday last week, and the 600 who attended this year's annual meeting of the Hospital Needlework and Linen Guilds, which was given order of precedence, had an agreeable experience. Lady Ninian Crichton-Stuart presided, and in the course of her speech remarked that "in the past the guilds have supplied all the linen required, but we shall require additional linen now because of the opening of the new wards, and we hope greater assistance will be forthcoming." The reports of Mrs. Henry Lewis and Mrs. Lynn Thomas, presidents respectively of the needlework and linen sections, revealed a substantial amount of work done for the upkeep of this important department. Colonel Bruce Vaughan paid a graceful tribute to her ladyship for her personal interest in the institution, and related that she rarely came to Cardiff without visiting the hospital to encourage those who worked there and to cheer the patients. The vote of thanks was seconded by Dr. H. Vachel as chairman of the medical board, and General Lee, the chairman of the board of management, said a few kind words of gratitude to all who had contributed to what is regarded as a successful year of work.

### HOSPITAL FOUNTAINS.

An additional interest was added to the above proceedings by an unusual function, the opening of the "Samuel Memorial" Fountain, which followed immediately after the meeting of the guilds. The central figure of the fountain represents Moses lifting up the serpent, and the inscription reads: "For love and remembrance of Hurman Samuel, who died in his sixtieth year, October 1910, this fountain was presented to the King Edward VII.'s Hospital, Cardiff, by his brothers and son." Mrs. Jack Samuel, of London, inaugurated the ceremony by turning on the fountain, and the gift was formally received by the Chairman of the Board of Management, Major-General Lee. Responding on behalf of Mrs. Samuel, Mr. Isaac Samuel said: "The Samuel family have been associated with this city for over half a century and my brothers and sisters were born and bred here. We recognise that they owe something to the town of their birth. We hail with cheerfulness any opportunity of fulfilling this debt. This is the eighth fountain given to the city by my brothers and myself. But with each gift there is a touch of grief and regret, for in giving these fountains as memorials each one, unfortunately, marks a further gap in our family circle. Bereavements are painful to recall, but in taking this method of memorising them there is the satisfaction that others gain where my immediate family loses. A strong advocate of temperance once told my brother-in-law, who resides in this city, that the drinking fountains to be seen in Cardiff are a stronger and more powerful force in the cause of temperance than any speech he had made or ever would make in its favour. If this is so, we have already reaped a great reward." The pleasant afternoon ended with tea on the children's lawn, where a musical programme was provided by the Cardiff Post Office Band under the conductorship of Mr. Paul Draper.

### THE WINNING DESIGN FOR THE DECORATION OF MIDDLESEX HOSPITAL.

The Committee of the Exhibition of Designs for Mural Painting and for the Decoration of Institutions, of which Mr. D. S. MacColl, keeper of the Wallace Collection, is chairman, and which closed at Crosby Hall, Chelsea, last Saturday, announces the result of the competition for the mural decoration of Middlesex Hospital. A prize of £50 has been awarded to Mr. Donald MacLaren, according to the original condition which Mr. Edmund Davis laid down that, in the event of a design being accepted and not approved by the Middlesex Hospital, the sum of £50 would be given in lieu of the execution of the work. It is, perhaps, advisable to add that though the winning design is not to be carried out, the fact of holding the competition at all has done a great deal for the cause of mural decoration and for acquainting artists with a hospital's requirements in this respect.

### THE FUTURE OF ENGLISH WALL-PAINTING.

Owing to the great interest shown in the Exhibition of Designs for Mural Paintings, which, as we announce above, nominally closed last Saturday, we understand that it will remain open till to-day (June 29). The Exhibition, apart from its practical results, is noteworthy as a considered attempt to force the attention of a distracted public on mural decoration, which, it is safe to repeat, is coming into fashion again in hospital work. The question is as much one for the superintendent as it is for the architect, and probably the not wholly satisfactory batch of designs at the Crosby Hall Exhibition is due to a want of mutual understanding of the two authorities chiefly concerned. The superintendent or secretary is naturally influenced by his perennial considerations of cost and cleansibility; to say nothing of a possible inexpertness in æsthetics; while the architect is often bothered by "a site" which offers technical artistic difficulties not easily understood by those whose profession is not that of architecture, or whose occupation is not that of painting. In truth, mural decoration of public buildings is somewhat of a revival, and just as nineteenth-century painting gained enormously from the return to the primitives which we call the pre-Raphaelite movement, and as some of the best English prose of the same period is that of William Morris, who took up literature and house decoration at the point to which it had been carried in the thirteenth century (in both cases the highest point the sections of art with which he was concerned had yet reached), so the future of English wall-painting must turn back and pick up to the till-lately obsolescent tradition. For the revival of any art is always a return to tradition. Hospital secretaries as well as the general public may well take to heart Professor Selwyn Image's recent pronouncement on this subject: "Do not treat wall-painting as a sort of bank-holiday lark"; and what architects engaged on mural decoration of hospital buildings must not be allowed to forget was pointed out to them in these Notes on April 20. Hospital secretaries not too much pressed for want of money, and there are a few as we all know in spite of the advertisements, should thank Mr. Walter Crane for his capital suggestion that "the money now spent on presentation portraits" might be spent with advantage on "presentation frescoes." On occasion, too, a mural painting might be offered as a substitute for naming a cot or endowing a bed to intending but unimaginative benefactors.

### THE KING AND LEICESTER INFIRMARY.

The announcement that his Majesty the King has conferred royal recognition upon the Leicester Infirmary has given great satisfaction to the local supporters of the institution which is held in high esteem by them. The fact, too, that the royal recognition comes at a time when efforts are being made to raise funds for the reconstruction of the Children's Hospital attached to the institution should stimulate these supporters quickly to subscribe the £8,500 required. So much has been done under the chairmanship of Sir Edward Wood to make the institution efficient at an expenditure of £100,000 that the small sum now asked for ought, and doubtless will, be raised if Leicester and the county are to live up to their great reputation for upholding charitable enterprise. The reconstruction scheme will add 22 cots to the capacity of the hospital, making 66 in all. A reference to the report of last year shows that the accounts are kept separate from those of the infirmary, but that the income was under £1,000. To maintain efficiently a Children's Hospital of 66 cots costs nearly £5,000 per annum, and we hope the Leicester public will see to it that the Children's Hospital receives a much greater measure of support than it has done. Sir Edward Wood and the Governors of the Leicester Royal Infirmary are, however, to be heartily congratulated on the honour conferred upon their institution, in the advance of which the administrative genius of Mr. Harry Johnson, the house governor and secretary, has been an example to hospital workers everywhere.

### A MAYOR'S WIT AT THE HEALTH CONFERENCE.

The Health Conference and Exhibition at the Royal Horticultural Hall, Westminster, the exhibits of which we summarised last week, was opened on Monday by the Mayor of Westminster. Professor Kenwood explained that the object of the Exhibition was to inform the public of the various provisions which were yearly being made in the interest of the public health. The Mayor of Kensington, in a witty and well-informed speech, reminded the company that it was far easier for a doctor to persuade a man to have his appendix removed than to sleep with his bedroom window open. Will Mr. Bernard Shaw please note?

### "THE HOSPITAL" AND RED CROSS ACTIVITIES.

Monday last was the Festival of St. John Baptist, and the Archbishop of York, Prelate of the Order of St. John of Jerusalem in England, preached the anniversary sermon at the annual commemoration service, which was held at the Priory Church of St. John, Clerkenwell. Afterwards Lord Knutsford, Sub-Prior of the Order, presided at the general assembly. The Senior Knight of Justice and Honorary Bailiff, Sir John Furley, C.B., spoke on his recent visit to Washington to represent the Order at the ninth International Conference of Red Cross Societies. He and the other representative, Colonel Guy Carleton Jones, Director-General of the Medical Services of the Canadian Forces, also a Knight of Grace of the Order, were the only representatives of England, except the Army medical officers detailed by the War Office. Sir John Furley stated that the business of the meeting included a variety of subjects which have been grafted on to the legitimate object of Red Cross work, and this tendency, which is not confined to our country, he ventured to criticise in a paper he read on "A National Red Cross Society and Affiliated Association."

### SIR JOHN FURLEY'S SUGGESTIONS.

It is interesting to note that Sir John Furley's remarks on this subject are made but a few days after our article in The Hospital last week, which explained critically the gist of the position and suggested the inevitable lines of reform. A complete field hospital under canvas and an exhibition of first-aid and hospital appliances attracted much attention at Washington. At the Washington Conference Sir John Furley said that soon after the establishment in England by the Order of St. John of the St. John Ambulance Association, an organisation to teach men and women to administer first aid in civil life, it was considered necessary to offer inducements to those who had gained certificates of proficiency, and so he made a tour of inspection of the English centres, and promised the pupils that, if we should be involved in war, the military authorities would accept the assistance of those of them who were competent as adjuncts to the Royal Army Medical Corps. Sir John emphasised his opinion that an arrangement in which *esprit de corps*, discipline, and efficiency were maintained by active usefulness during peace was the best and most practical in relation to the possible demands of war. Governments could only recognise one Red Cross Society for each nation; but to this society other associations might become affiliated in time of peace, and connected with the central body by representatives in such a manner that their mobilisation might take place simultaneously with that of the National Army. A central Red Cross committee would be fully employed in the management of its funds and in the creation and supervision of its administrative machinery; and it might leave to affiliated societies the task of finding, training, and keeping together the necessary personnel.

### A CORONER IN THE PROFESSORIAL CHAIR.

Dr. William Sellers, M.D., has been appointed Professor of Forensic Medicine in succession to the late Dr. Dixon Mann at the Victoria University, Manchester. Dr. Sellers, who is a graduate of London University, after engaging in general practice, became Medical Officer of Health for Radcliffe for thirteen years, when he retired from practice only to be called to the Bar. For nine years he was Manchester's deputy coroner, and now holds that post for the Salford district.

### THE MORAL FOR A HOSPITAL AT LOGGERHEADS.

A dispute is in progress between the Board of Management of the Mansfield Hospital and the Guardians, whose repeated criticisms of the hospital's management have culminated in a charge of inconsistency. The hospital's latest offence in the eyes of the Guardians is a sequel to its recent refusal by resolution to admit patients whose injuries are due to attempted suicide by accepting a case of this sort last week. One of the Guardians, Mr. J. Ashley, attributed this admission to the fact that "the patient came from a big house." Another, Dr. Martyn, of Sutton, said that it was unfortunate that there was no qualified person at the hospital in whose hands discretion in applying the resolution could be placed. The reason apparently offered by the hospital: that patients would complain if cases of attempted suicide were admitted, was stigmatised by Mr. Ashley as a rank libel. Whereupon, Mr. F. P. Cook, who presided at the meeting, pointed out, first, that at present the hospital was not possessed of an isolation ward, though he hinted such might yet be included in the present extension; and, secondly, that a house surgeon would be appointed before long, with the result that "many of these difficulties would disappear." The quietness of his speech is more impressive than the style of his predecessor, and on the bare facts which we have stated the argument of Mr. Cook will impress most hospital workers as being on the right lines. But the moral for the hospital is—"Appoint a house surgeon."

### HOSPITAL CONCERTS IN BIRMINGHAM.

Only last August we called attention to the system of hospital concerts in Germany, which drew an interesting letter from Mr. Alfred Whitham, the secretary of the Beckett Hospital Sunday Fund movement, in which he explained how the annual wakes of the Yorkshire villages had been converted by certain musical enthusiasts into a source of income for the Beckett Hospital, Barnsley, and, incidentally, we gathered, without destroying the spirit of these reunions. It is, therefore, interesting to record that what music has done for a Yorkshire hospital music is doing at Birmingham also, for on Thursday last week £450 was handed over to the Birmingham and Midland Hospital for Women, on behalf of the Birmingham and District Sunday Evening Concert and Lecture Committee. The concert secretary is Mr. G. A. Parker, who handed the cheque to the Lord Mayor, remarking as he did so that these concerts were now in their sixth year. The Lord Mayor, Mr. Alderman W. H. Bowater, like possibly many of our readers, acknowledged surprise at the largeness of the sum which the concerts had produced. The president of the concert committee is Mr. F. Mountjoy, who introduced his committee into the Lord Mayor's parlour, where the treasurer of the hospital, Mr. Martin, accepted the cheque from his lordship's hands. In thanking the concert committee, on behalf of the hospital, for their cheque, Mr. J. S. Nettlefold, the chairman, reminded those present that twelve months had passed since the hospital's committee had taken what some thought the unwise risk of carrying on the Maternity Hospital when it was threatened with closure. It was, he said, certainly in need of money. It should now have the additional satisfaction, in accepting this splendid gift, of feeling that it is indirectly doing something to encourage the most typical of the fine arts, and the one, too, which in England still involves the greatest discomfort and the most outrageous expense, to the amazement of our Continental neighbours and to our own discredit.

### SIR FRANK WILLS.

The honour of knighthood which falls to the lot of the Lord Mayor of Bristol, Mr. Frank W. Wills, is noteworthy, inasmuch as he has in his professional capacity as an architect done institutional work, including extension and renovation, in his native city. He comes from a family whose various members have contributed for many years past to hospitals.

### FORTY-SEVEN PERSONS IN A PADDINGTON HOUSE.

A hideous example of overcrowding was revealed at the Marylebone Police Court last week, when the Paddington Borough Council prosecuted under the Housing and Town Planning Act, 1909. It appeared that a nine-roomed house known as 17 Desborough Street, off the Harrow Road, was occupied by thirty-five children and twelve adult persons. Dr. R. Dudfield, the Medical Officer of Health, stigmatised the place as unfit for human habitation, and the irony of extreme poverty is seen in the fact that the defendants were five of the tenants who were indicted for refusing to quit after a closing order had been made! The five stated that collectively they had twenty-nine children, and it was left for the sanitary inspector, Mr. Wingfield, to add that another tenant had gone there with six children, making a total of forty-seven in all. An order was made by Mr. Paul Taylor for the defendants to quit the house within fourteen days.

### BY-WAYS OF HOSPITAL FINANCE.

A meet of cyclists organised by the Woodford Cyclist Meet Association in aid of the funds of local hospitals was held for the fifteenth year in succession last Saturday. The weather was brilliant and hundreds of cyclists from the surrounding districts, many of whom were in fancy costume, assembled at Wanstead, and after the prizes had been awarded for various classes the procession made a tour of Woodford and district. At dusk the cycles were illuminated for the return ride. The lay public uninitiated in hospital finance would probably be surprised to learn that this carnival since its inauguration has raised a sum of about £5,000 on behalf of local institutions by collections previous to and on the day of the annual meet.

### RESIDENTIAL SCHOOLS FOR CONSUMPTIVE CHILDREN.

The result of a conference held at Bradford between the Corporation Health Committee and the Elementary Education Committee has resulted in a resolution being passed to the effect that residential schools for tuberculous children are essential. This decision will surprise no one who understands the preventive methods of anti-phthisis effort, and was only to be expected from Bradford, where so many important schemes of late on this and allied measures have originated. Our readers will remember an account which we published last summer of an open-air school for tuberculous children that was opened in Paddington. It contained the statement that much of the good which the school was capable of doing by keeping the children of very poor people in the open air and under hygienic supervision was impaired by their return to disastrous home surroundings at nightfall. It was hoped that meals might be provided at the school in spite of the parrot cry of unthinking people that it would pauperise those who were already beyond that condition, but the authorities were bound to admit that a residential school would make genuine progress more certain. The truth of this seems to have been grasped by Bradford, which is to be congratulated for its decision to undertake the above experiment.

### MEDICAL PRACTITIONERS AND PROPRIETARY PRODUCTS.

The Select Committee on Proprietary Medicines, as The Hospital forecast on April 20, has become acquainted with the fact that some medical practitioners have come to prescribe proprietary products, the composition of which is not publicly known, and is endeavouring to find its explanation. The most important part of the cross-examination of Dr. Alfred Cox, medical secretary of the British Medical Association, which occupied several sittings, was that which had reference to the position of medical men in relation to proprietary articles. When one of the members of the Committee, Mr. W. S. Glyn-Jones, read an advertisement for a proprietary emulsion which appeared in the official organ of the British Medical Association, Dr. Cox observed, "That is as mild as milk compared with the statements which appear in the daily papers." There is, of course, a vast difference between the products advertised to medical men and those advertised to the public, but unfortunately the case against secrecy in medicine is not helped by the fact that it is sometimes unwittingly encouraged. Asked whether his evidence did not go to show that, while there are some medicines which the Association would desire to suppress, there are others which need not be suppressed, Mr. Cox replied, "Undoubtedly." The witness expressed the hope that when fourteen million working people have their wants attended to by doctors under the Insurance Act, the sale of patent medicines would decrease very considerably.

### THEATRE UNITS IN MODERN COTTAGE HOSPITALS.

A new up-to-date operating theatre, with a sterilising room and a recovery ward, was opened on Saturday week at the Market Drayton Cottage Hospital by Mrs. Monro Walker. The extension, which is a memorial to King Edward VII., was built at the expense of Mr. J. Monro Walker, of Pell Wall, Market Drayton, and has been formally handed over by him to the trustees of the institution. At the close of the proceedings, which, at the express wish of Mr. Walker, were of a purely private character, a sum of over £20 was subscribed by those present towards the furnishing of the rooms. We are glad to note this unobtrusive function, which is typical of the best side of the voluntary spirit, for there can be no doubt that a well-cared-for cottage hospital is an influence for good in any county which it would be hard to overestimate.

### THIS WEEK'S DRUG MARKET.

The usual fortnightly auction of drugs in Mincing Lane will be held on the day fixed, notwithstanding that the labour troubles at the docks are not yet settled. The process of unloading and forwarding goods is not yet working with its normal smoothness, but the position has vastly improved and in a week's time there is not likely to be much inconvenience. The Drug market has been exceedingly dull and quiet, and there have been very few price changes since the noteworthy advance in the price of iodine and iodides announced last week. Rhubarb still tends upwards in value; citric acid is rather dearer, and orris root is scarce. Opium is in a peculiar and uncertain position, and it would perhaps be advisable for buyers of this drug, as well as of morphine and codeine, to postpone their purchases for the present. A further reduction in the price of cocaine would not come as a surprise.

### TO OUR READERS.

Contributions are specially invited from any of our readers to these columns. They should deal with topical subjects and news. They must be authenticated for the information of the Editor only. The minimum payment if published is 5s. There is no hard-and-fast rule as to space, but notes of about twenty lines in length are preferred.

